# Fine-Grained Mapping of Cortical Somatotopies in Chronic Complex Regional Pain Syndrome

**DOI:** 10.1523/JNEUROSCI.2005-18.2019

**Published:** 2019-11-13

**Authors:** Flavia Mancini, Audrey P. Wang, Mark M. Schira, Zoey J. Isherwood, James H. McAuley, Giandomenico D. Iannetti, Martin I. Sereno, G. Lorimer Moseley, Caroline D. Rae

**Affiliations:** ^1^Computational and Biological Learning, Department of Engineering, University of Cambridge, Cambridge CB2 1PZ, United Kingdom,; ^2^Department of Neuroscience, Physiology and Pharmacology, University College London, London WC1E 6BT, United Kingdom,; ^3^Neuroscience Research Australia, Sydney, New South Wales 2031, Australia,; ^4^Faculty of Medicine and Health and Faculty of Health Sciences, University of Sydney, Sydney, New South Wales 2145, Australia,; ^5^School of Psychology, University of Wollongong, Wollongong, New South Wales 2522, Australia,; ^6^School of Medical Sciences, University of New South Wales, Sydney, New South Wales 2052, Australia,; ^7^Neuroscience and Behaviour Laboratory, Istituto Italiano di Tecnologia, Rome 00161, Italy,; ^8^Department of Psychology, University College London, London WC1E 6BT, United Kingdom,; ^9^Department of Psychology, San Diego State University, San Diego, California 92182, and; ^10^IMPACT in Health, University of South Australia, Adelaide, South Australia, Australia

**Keywords:** chronic pain, fMRI, plasticity, S1, somatosensory cortex

## Abstract

It has long been thought that severe chronic pain conditions, such as complex regional pain syndrome (CRPS), are not only associated with, but even maintained by a reorganization of the somatotopic representation of the affected limb in primary somatosensory cortex (S1). This notion has driven treatments that aim to restore S1 representations in CRPS patients, such as sensory discrimination training and mirror therapy. However, this notion is based on both indirect and incomplete evidence obtained with imaging methods with low spatial resolution. Here, we used fMRI to characterize the S1 representation of the affected and unaffected hand in humans (of either sex) with unilateral CRPS. The cortical area, location, and geometry of the S1 representation of the CRPS hand were largely comparable with those of both the unaffected hand and healthy controls. We found no differential relation between affected versus unaffected hand map measures and clinical measures (pain severity, upper limb disability, disease duration). Thus, if any map reorganization occurs, it does not appear to be directly related to pain and disease severity. These findings compel us to reconsider the cortical mechanisms underlying CRPS and the rationale for interventions that aim to “restore” somatotopic representations to treat pain.

**SIGNIFICANCE STATEMENT** This study shows that the spatial map of the fingers in somatosensory cortex is largely preserved in chronic complex regional pain syndrome (CRPS). These findings challenge the treatment rationale for restoring somatotopic representations in complex regional pain syndrome patients.

## Introduction

Chronic pain is a highly common and debilitating disorder that can be associated with functional and morphological changes in the brain. For instance, it has long been thought that some severe chronic pain conditions, such as complex regional pain syndrome (CRPS), are not only associated with, but even maintained by, maladaptive topographic changes in the primary somatosensory cortex (S1) (Maihöfner et al., 2003, 2004). MEG and EEG studies have suggested that the representation of the CRPS hand in S1 is abnormally smaller than the cortical representation of the healthy hand (Juottonen et al., 2002; Maihöfner et al., 2003; Pleger et al., 2004; Vartiainen et al., 2008, 2009). The notion of S1 reorganization has been central to our understanding of the condition (Marinus et al., 2011) and has driven physiotherapy interventions aimed at restoring sensorimotor representations of CRPS limbs, such as mirror-visual feedback (McCabe et al., 2003; Smart et al., 2016) and sensory discrimination training (Pleger et al., 2005; Moseley et al., 2008a). Here, we revisit the notion of S1 reorganization with the better tools that modern fMRI currently offers: high spatial resolution and phase-encod methods that provide reliable and unbiased measures of the cortical somatotopy of the hand (Mancini et al., 2012; Sanchez-Panchuelo et al., 2012; Kolasinski et al., 2016a).

The somatotopy of the full hand has never been characterized in CRPS patients. In all previous studies on CRPS, the size of the hand map was estimated both indirectly and incompletely, by measuring the Euclidean distance between activation loci of the thumb or index finger relative to that of the little finger. These Euclidean measures are problematic because they disregard that the central sulcus is not flat and they do not provide a direct measure of map area.

A more reliable fMRI method for studying cortical topographic representations is based on phase-encoded mapping, which reveals the spatial preference of cortical neural populations (Sereno et al., 1995; Silver and Kastner, 2009; Sereno and Huang, 2014). This method involves delivering a periodic sensory stimulus to different portions of the receptive surface and evaluating which voxels selectively respond to the spatial frequency of the stimulation. Voxels sensitive to the stimulus respond when the stimulus is delivered at the preferred spatial location, and they decay as the stimulus moves away (Chen et al., 2017). The response phase angle, extracted using a Fourier transform (Mancini et al., 2012), indicates the location preference for each voxel; in other words, the position of the receptive fields of the population of neurons sampled by the voxel.

Using phase-encoded mapping, we provide the first complete characterization and quantification of the representation of the fingers (i.e., with exclusion of the thumb) in patients with chronic and unilateral CRPS to the upper limb. We tested whether the S1 representation of the fingertips of the affected hand was different from that of the healthy hand of CRPS patients and from controls in terms of its spatial extent, location relative to the central sulcus, and geometry (i.e., variability of the map gradients).

## Materials and Methods

### 

#### 

##### Participants.

We recruited 20 adults with unilateral CRPS to the upper limb (either right or left side) and 20 healthy controls of either sex, matched for age, gender, and handedness. Each participant gave written informed consent to take part in the study. All experimental procedures were performed in accordance with the Declaration of Helsinki and approved by both the Human Research Ethics Committee of the University of New South Wales (HC13214) and by the Human Ethics Committee of the South Eastern Local Health District (HREC 10/051). Inclusion criteria for control participants were as follows: (1) pain-free at the time of the study; (2) no prior history of a significant chronic pain, psychiatric or medical disorder; and (3) no history of substance abuse. Inclusion criteria for CRPS patients were as follows: (1) a diagnosis of unilateral CRPS to the upper limb or hand according to the Budapest research criteria (Harden et al., 2010); (2) CRPS duration >3 months; and (3) no history of substance abuse and no psychiatric comorbidities. Five of 40 participants were excluded from the study due to the following problems: MRI scanner failure, acquisition problems, and breach of eligibility criteria (a control participant reported pain to the wrist on the day of scan; a median nerve compression was subsequently diagnosed). The demographic and clinical information of the remaining sample (Controls: *n* = 17; CRPS to the left hand: *n* = 8; CRPS to the right hand: *n* = 10) is reported in Table 1.

**Table 1.** Demographic and clinical information of the study sample*^a^*

ID	Group	Age (yr)	Gender	CRPS duration (yr)	Incident at onset	Pain location	Location of other CRPS symptoms	Range of motion	Motor weakness	Tremor	Allodynia	Pain rating during scan	Mean pain rating over 2 d	Mean pain rating over 7 d	PPT left hand (kg/cm^2^)	PPT right hand (kg/cm^2^)	Laterality score
C1	Control	38.5	F	NA	NA	NA	NA	–	–	–	–	1	0	0	50.5	62.2	87.5
C2	Control	42.8	M	NA	NA	NA	NA	–	–	–	–	4	0	0	13.4	13.6	87.5
C3	Control	52.8	M	NA	NA	NA	NA	–	–	–	–	0	0	0	43.7	40.8	87.5
C4	Control	41.1	M	NA	NA	NA	NA	–	–	–	–	0	0	0	4.72	3.87	100
C5	Control	56.6	M	NA	NA	NA	NA	–	–	–	–	0	1	1	5.03	4.83	73.3
C6	Control	42.3	M	NA	NA	NA	NA	–	–	–	–	0	0	0	3.6	4.78	87.5
C7	Control	34.1	M	NA	NA	NA	NA	–	–	–	–	0	0	0	3.84	4.72	64.7
C8	Control	53.0	M	NA	NA	NA	NA	–	–	+	–	0	0	0	5.36	5.55	66.7
C9	Control	48.7	F	NA	NA	NA	NA	–	–	–	–	0	0	0	7.92	7.39	100
C10	Control	56.5	M	NA	NA	NA	NA	–	–	–	–	0	0	0	3.92	3.27	83.3
C11	Control	46.9	M	NA	NA	NA	NA	–	–	–	–	0	0	0	5.58	5.73	100
C12	Control	38.4	M	NA	NA	NA	NA	–	–	–	–	0	0	0	3.7	4.75	100
C13	Control	47.8	M	NA	NA	NA	NA	–	–	–	–	0	0	0	5.6	5.53	−100
C14	Control	25.2	F	NA	NA	NA	NA	–	–	–	–	0	0	0	4.41	4.88	100
C15	Control	19.9	M	NA	NA	NA	NA	–	–	+	–	0	0	0	5.4	5.14	12.5
C16	Control	49.2	M	NA	NA	NA	NA	–	–	–	–	0	0	0	6.52	7.71	100
C17	Control	69.4	M	NA	NA	NA	NA	–	–	–	–	0	0	0	3.83	3.42	100
P1	CRPS to left hand	42.4	M	1.2	Wrist fracture	L wrist, hand	L UL	+	+	+	+	7	7	8	1.9	12.2	50
P2	CRPS to left hand	42.8	M	0.9	Wrist injury	L hand	L UL	+	+	–	+	8	4	4	10.6	12.3	83.3
P3	CRPS to left hand	55.6	M	0.5	Frozen shoulder	L wrist, hand	L UL	+	+	+	–	9	8	9	2.06	4.13	83.3
P4	CRPS to left hand	45.3	M	3.8	Hand surgery	L wrist, hand	L UL	+	+	+	+	7	8	8	1.8	2.55	4.3
P5	CRPS to left hand	66.7	M	0.4	Hand injury and infection	L wrist, D1, D2	L hand	+	+	+	+	2	9	7	0.88	4.31	73.9
P6	CRPS to left hand	47.8	M	4.1	Hand surgery	L D1, D4, D5	L UL	–	+	+	+	0	3	5	2.9	3.89	100
P7	CRPS to left hand	41.9	M	14.9	Hand trauma injury	L forearm, wrist, hand	L UL	+	+	+	+	7	6	6	0.33	3.55	100
P8	CRPS to left hand	29.2	M	1.8	Hand injury	L UL	L UL	+	+	+	+	6	7	5	0.76	2.06	−100
P9	CRPS to right hand	53.4	M	4.5	Shoulder injury	R UL	R UL	–	+	–	+	9	6	7	49.2	15.5	100
P10	CRPS to right hand	38.1	M	0.4	Hand and wrist injury	R wrist, hand	R UL	+	+	+	+	8	6	5	6.925	2.925	85.7
P11	CRPS to right hand	51.6	M	0.4	Hand injury	R hand	R UL	+	+	+	+	2	3	3	4.77	3.53	89.5
P12	CRPS to right hand	34.7	M	2.9	Hand fracture	R wrist, hand	R UL	+	+	+	+	7	6	6	3.82	1.3	73.3
P13	CRPS to right hand	48.7	F	2.6	Wrist fracture and surgery	R wrist, hand	R wrist, hand	+	+	+	+	8	7	7	12.59	4.94	100
P14	CRPS to right hand	46.7	M	7.5	Shoulder injury	R UL	R UL	–	+	+	+	5	3	3	2.37	1.06	66.7
P15	CRPS to right hand	56.8	M	14.6	Arm injury	R UL	R UL	+	+	+	+	10	3	3	3.07	0.9	100
P16	CRPS to right hand	26.6	M	1.9	Wrist fracture	R wrist, D2, D3, D4	R UL	+	+	+	+	7	6	3	3.1	0.98	−66.7
P17	CRPS to right hand	21.4	F	2.6	Wrist and hand trauma injury with surgery	R wrist, hand	R hand, arm	+	+	+	+	0	4	5	4.79	3.84	100
P18	CRPS to right hand	44.9	M	18.3	Road traffic accident	R UL	R UL	+	+	+	+	9	8	8	3.63	2.45	64.7

*^a^*Pain location indicates the self-reported location of pain sensations. Location of other CRPS symptoms' describes the location of all other sensory and motor symptoms (including allodynia). D1-D5, Affected digit; hand, whole hand (including all digits); UL, whole upper limb (shoulder, arm, wrist, hand, fingers). Range of motion, motor weakness, tremor, allodynia: −, No abnormality; +, presence of a symptom. Intensity of pain to the upper limb during scans was evaluated on a Likert scale from 0 (no pain) to 10 (worst pain imaginable). The laterality score is derived from the Edinburgh Handedness Inventory and ranges from −100 (left-hand dominant) to 100 (right-hand dominant).

##### Clinical evaluation.

Patients were clinically evaluated according to the Budapest research criteria (Harden et al., 2010) by a blinded researcher of the team on the first session of the study to confirm that the research criteria were met. As part of the clinical and diagnostic assessment of CRPS, we assessed pressure pain thresholds (PPTs; kg/cm^2^) using a digital pressure algometer (Wagner Instrument) on two sites of each hand: the thenar eminence and the third proximal interphalangeal joint. Pain intensity was also rated using an 11 point Likert scale, where 0 corresponded to “no pain” and 10 indicated “the worst pain imaginable, like a red hot poker through your eye.” The intensity of spontaneous pain in the upper limb was rated in all patients immediately before, during, and after the imaging session. Patients were also asked to rate the average pain intensity experienced both over the 48 h and the 7 d preceding the MRI session. Two control participants reported discomfort and mild to moderate postural pain to the upper limb during the scanning session (Table 1). Furthermore, the *Quick*Dash (Disabilities of the Arm, Shoulder and Hand) questionnaire was administered to all participants; the *Quick*Dash measures physical function and symptoms in people with musculoskeletal disorders of the upper limb (Kennedy et al., 2011).

##### Stimuli.

We used a customized stimulus (polypropylene probe with a rounded tip) because CRPS skin physiology and symptoms (hand dystonia, pain) preclude the use of conventional and automated mechanical stimulation for the prolonged time required for phase-encoded mapping of the fingertips (∼40 min). For example, hand dystonia makes it difficult to target the same skin regions with air-puffs throughout the imaging session; this would have resulted in scan quality deterioration or early scan termination. CRPS-related hyperhidrosis (i.e., excessive sweating) precludes the use of electrical and vibrotactile stimulation for long periods of time.

All control participants reported the stimulus as being clearly detectable, neither painful nor unpleasant, and similarly intense on the different fingers of the two hands. All patients described the sensation that was elicited by stimulation of the unaffected fingers, in similar terms to those used by the control participants. Patients described the sensation that was elicited by stimulation of the affected fingers in a variety of ways; “burning,” “tingling,” “pain,” “brushing like with a sharp object,” “horrible,” “itchy,” “scraping,” “like a needle prick,” “electric shooting pain.” These terms are consistent with the clinical phenomenon of allodynia.

Participants did not report systematic differences in stimulus perception across the fingertips of the same hand. Pain intensity fluctuates over time in most chronic pain conditions (including CRPS), even despite highly controlled and reproducible stimulation (Foss et al., 2006). However, such fluctuations are unlikely to confound our measures of cortical somatotopy. Indeed, our analysis method allowed to dissect the magnitude of the brain responses from their spatial organization. All our analyses did not focus on the magnitude of the S1 responses, but on their spatial organization, which is not confounded by unavoidable fluctuations of perceived stimulus intensity in CRPS patients.

##### Experimental design.

Each participant laid supine inside the scanner bore with both hands palm upward. Participant's arms and hands were propped with cushions and pads to minimize movements. The stimulus consisted of periodic stimulation of the fingertips of both hands. In each stimulation cycle, the tips of the index, middle, ring, and little fingers were successively stimulated using a customized probe (see below). Each fingertip was stimulated for 6 s, and each cycle (four fingers × 6 s = 24 s) was interleaved by 6 s of rest. Twelve cycles were administered in each of the four consecutive functional runs (∼10 min each). Two trained experimenters stimulated the tips of homologous fingers of the right and left hands simultaneously. The experimenters received auditory cues through headphones, synchronizing the location and timing of each stimulus. The thumb was not stimulated to reduce scanning time and due to practical difficulties in stimulating the thumb in succession to the other fingertips (patients could not keep the hand open flat for prolonged periods of time).

**Figure 1.**
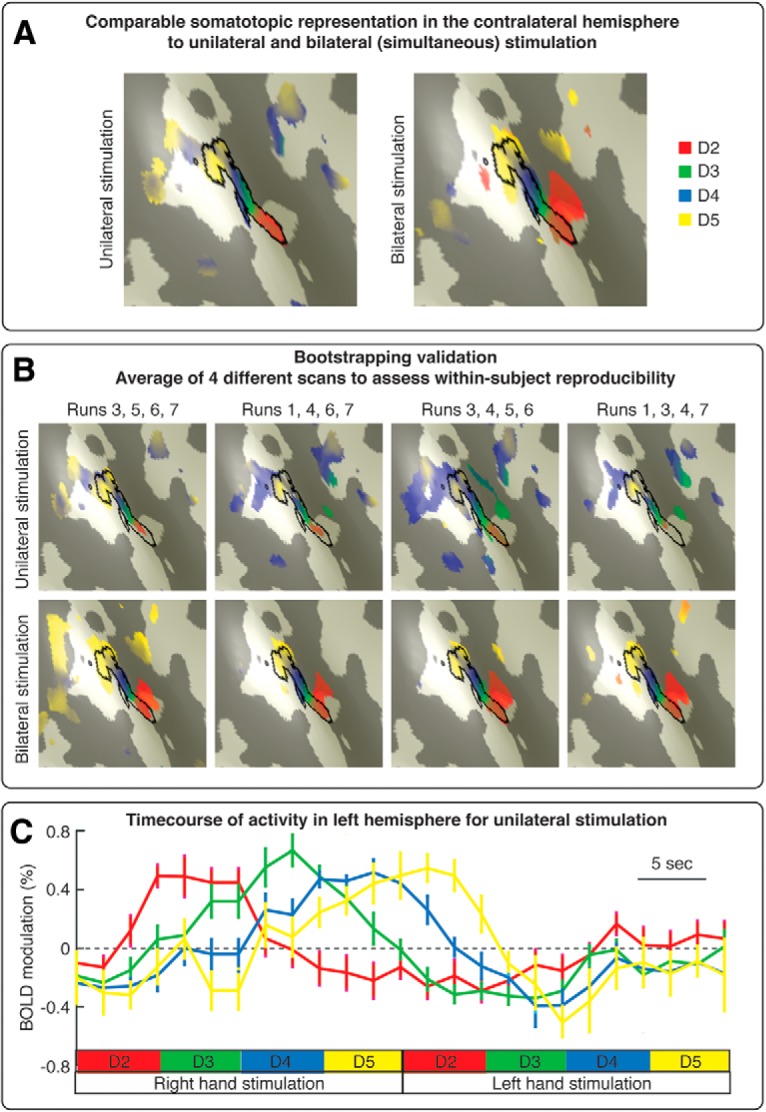
Preliminary results that guided the design of the finger mapping protocol. ***A***, Comparable somatotopic representation in the contralateral S1 to unilateral and bilateral finger stimulation, at within-subject level. The map of the fingertips (d2–d5) in contralateral S1 was strikingly similar in a condition in which we stimulated the fingertips of one hand at time and in another condition whereby we stroked homologous fingertips of both hands simultaneously. ***B***, Bootstrapping validation. We validated the results shown in ***A*** using a bootstrapping approach. Seven functional runs per condition (unilateral stimulation, bilateral stimulation) were collected in a single participant, in multiple scanning sessions. We selected, both recursively and randomly, 4 runs among the 7 collected per condition and averaged results across these 4 runs to assess intraindividual map reproducibility. The maps of the fingertips were highly reproducible in both unilateral and bilateral stimulation conditions. ***C***, Time course of activity in the left hemisphere during unilateral fingertip stimulation. Percent modulation of BOLD response in the left S1 induced by periodic stimulation of the fingertips of the right hand and left hand. We did not observed a spatially tuned activation of the left S1 induced by left-hand stimulation.

Our choice of bilateral stimulation was motivated by the need to map the fingertips of both hands in a single imaging session (several patients traveled from distant regions in Australia). Importantly, our choice was grounded on neuroscientific evidence that there are extremely limited trans-callosal connections between the hand representations of S1 in the primate brain (Jones and Hendry, 1980; Killackey et al., 1983). Indeed, a previous fMRI study reported a great similarity between the S1 map of the hand elicited by unimanual versus bimanual finger movements (Kikkert et al., 2016, their Fig. 2*D*,*E*). This is further confirmed by our preliminary imaging data, in which we found that unilateral versus bilateral fingertip mapping yielded both greatly similar and highly reproducible fingertip maps in S1 (Fig. 1).

We note that some studies have reported an inhibitory response to unilateral hand movements in ipsilateral S1 (Hlushchuk and Hari, 2006; Lipton et al., 2006; Klingner et al., 2011; Diedrichsen et al., 2013). The deactivation of ipsilateral S1 is most likely mediated by an input that ascends the contralateral pathway to a higher-order cortical area, crosses the midline through the corpus callosum, and is then fed back to area 3b in S1 (Lipton et al., 2006; Tommerdahl et al., 2006). Ipsilateral activations and deactivations in S1 during unilateral movements are diffused and not somatotopically specific (Helmich et al., 2005; Reed et al., 2011; Ann Stringer et al., 2014; Tal et al., 2017). Even if they modulate the amplitude of the S1 response (which is unlikely and also not of interest here), there is no evidence that they affect the spatial (somatotopic) organization of the contralateral responses (Reed et al., 2011; Ann Stringer et al., 2014; Kikkert et al., 2016; Tal et al., 2017). Importantly, ipsilateral activity of sensorimotor cortex vanishes during bimanual movements (Diedrichsen et al., 2013) and during passive somatosensory stimulation (Berlot et al., 2019).

For these reasons, we considered bilateral finger stimulation as a resource-efficient method to map the S1 somatotopy of the fingers of both hands in a single imaging session, thus boosting recruitment and compliance of CRPS patients.

##### MRI acquisition.

Echoplanar images (1.5 mm^3^ isotropic resolution, 183 volumes/run, 32 axial slices, matrix size of 128 ×128, FOV 192 × 192, SENSE factor of 2.4, flip angle = 82°, TR = 2 s, TE = 25 ms, no partial Fourier) were collected in four runs on a Philips Achieva TX 3T MRI scanner using a 32-channel head coil. FreeSurfer (https://surfer.nmr.mgh.harvard.edu/) was used to reconstruct the cortical surface for each subject from a structural T1 image (0.727 × 0.727 mm^2^ in-plane, 0.75-mm-thick slices, 250 slices, flip angle = 8°, TR = 6.318 ms). In 4 subjects (P7, P8, P17, C15), structural T1 images were corrected for nonuniform intensity using the AFNI's tool 3dUnifize (https://afni.nimh.nih.gov), before surface reconstruction, because these images contained shading artifacts that could have affected segmentation.

##### First-level MRI analyses.

All first-level analyses were performed by a researcher blinded to the group condition (right CRPS, left CRPS, control). The first 3 volumes were discarded from all analyses. Functional series were aligned and motion-corrected using the AFNI program 3dvolreg. Using this as a starting point, functional-to-high resolution alignment was then refined using manual blink comparison using an adaptation of Freesurfer's TkRegister implemented in csurf (http://www.cogsci.ucsd.edu/∼sereno/.tmp/dist/csurf). After linear trend removal, aligned data from the four runs were raw-averaged, and then analyzed using a fast Fourier transform, computed for the time series at each voxel fraction (vertex): this resulted in complex valued signals with the phase angle and magnitude of the BOLD response at each voxel. The phase angle is the measure of interest here because it reflects the spatial preference of a given voxel. Both Fourier and statistical analyses were performed using csurf. No spatial smoothing was performed before statistical analyses. Very low temporal frequencies and harmonics (<0.005 Hz) were excluded because movement artifacts dominate responses at these frequencies, a procedure virtually identical to regressing out signals correlated with low-frequency movements. High frequencies up to the Nyquist limit were allowed (i.e., half the sampling rate); this corresponds to no use of low pass filter. For display, a vector was generated whose amplitude is the square root of the *F* ratio calculated by comparing the signal amplitude at the stimulus frequency to the signal at other noise frequencies and whose angle was the stimulus phase. To minimize the effect of superficial veins on BOLD signal change, superficial points along the surface normal to each vertex were disregarded (top 20% of the cortical thickness).

The *F* ratio was subsequently corrected at *p* < 0.01 using a surface-based cluster correction for multiple comparisons as implemented by surfclust and randsurfclust within the csurf FreeSurfer framework (Hagler et al., 2006). The Fourier-transformed data were then sampled onto the individual cortical surface. Using this statistical threshold, we cut a label containing all vertices that showed a significant periodic response to finger stimulation (see one example in Fig. 7*A*), localized within S1 (i.e., within the boundaries of areas 3a, 3b, and 1, as estimated by the cortical parcellation tools implemented in Freesurfer). This label, or ROI, was used as the input for the analyses described in the next sections. The phase-encoded stimulation procedure that we used is designed to map the hand region across fingers, not within fingers (Sanchez-Panchuelo et al., 2012). Therefore, we could not derive accurate ROIs for each finger in isolation. This is because voxels that are activated by more than one finger are masked out. Furthermore, we did not derive ROIs for the different subdivisions of S1 because a precise and reliable parcellation of the cortical surface at single-subject level would require microstructural imaging.

In a few cases, we could not identify any ROI with a response to fingertip stimulation (no response to either fingertip stimulation), even at uncorrected *p* < 0.05: Subject P9, right hemisphere (patient with right CRPS); Subject P14, left hemisphere (right CRPS); Subject P15, left hemisphere (right CRPS); Subject P7, left hemisphere (left CRPS); and Subject P8, right hemisphere (left CRPS). These cases were treated as missing data in further analysis.

#### Statistical analysis

##### Evaluation of hand map area.

We calculated the surface area of the left- and right-hand maps, from each participant ROI. This was done after resampling the phase maps onto the original average brain volume, to control for interindividual variability in brain size. To increase statistical power, we flipped the data from the right hand CRPS group so that the affected side became the left hand/right hemisphere in all patients and then pooled these data. Upon testing for normality, we compared map area in the affected versus unaffected sides with both a frequentist and a Bayesian mixed-effects ANOVAs with a within-subject factor “side” (two levels: affected, unaffected) and a between-subjects factor “group” (two levels: controls, CRPS). Bayes factors (BFs) were classified and interpreted following the JASP guidelines for conducting and reporting a Bayesian analysis (van Doorn et al., 2019) (see Fig. 4).

##### Evaluation of hand map location.

We controlled for individual differences in brain morphology as follows. We first inflated each participant's cortical surface to a sphere and then nonlinearly morphed it into alignment with an average spherical cortical surface using FreeSurfer's tool mri_surf2surf (Fischl et al., 1999). This procedure maximizes alignment between sulci (including the central sulcus) while minimizing metric distortions across the surface. We resampled phase maps onto this average spherical surface (Freesurfer's fsaverage) and calculated the location of the centroid of the map on this average surface. We investigated whether the map centroid was different across sides and groups, in two ways.

First, we tested whether the distribution of spherical coordinates was different across conditions (side and group). As a basis for this comparison, we used the Fisher probability density function (Fisher, 1953), which is the spherical coordinate system analog of the Gaussian probability density function. This approach has been commonly used in the field of paleomagnetism and has also been applied for the analysis of direction data from diffusion tensor imaging (Hutchinson et al., 2012). We calculated the *F* statistics for the null hypothesis that sample observations from two groups are taken from the same population. The following equation was derived from Watson (Watson, 1956; Hutchinson et al., 2012) and used to compare two groups with *N*_1_ and *N*_2_ observed unit vectors and resultant vectors of length *R*_1_ and *R*_2_, respectively, as follows:

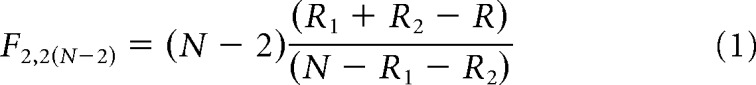
 where *N* = *N*_1_ + *N*_2_ and *R* is the length of the resultant vector for the pooled direction vector observations from both groups. The resultant vector sums of all observations, *R*_1_, *R*_2_, and *R*, are calculated as follows:

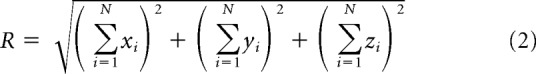
 where *x_i_*, *y_i_*, and *z_i_* are the coordinates of the map centroids for each participant.

We performed the following *F* contrasts, separately for each hemisphere: controls versus patients with right CRPS and controls versus patients with left CRPS (four *F* tests in total). The larger the value of *F*, the more different the two group mean directions. A *p* value was obtained using the appropriate degrees of freedom (2 and 2(*N* − 2), respectively) and critical probability level of 0.05. The *F* statistics for *H*_0_ (no difference) and *H*_1_ were used to calculate the BF for each contrast (Held and Ott, 2018, their Eq. 5) as follows:

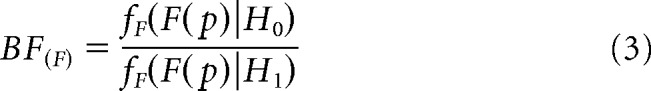
 The *F*-based BF_10_ is simply equal to 1/BF_(_*_F_*_)_.

As a complementary measure of map location, we computed the geodesic distance (in millimeters) between the map centroid and an arbitrary reference point located within the concavity of the central sulcus (displayed in Fig. 6*C*). Geodesic distances were statistically compared using both a frequentist and a Bayesian mixed-effects ANOVA with a within-subject factor side (two levels: affected, unaffected) and a between-subjects factor group (two levels: controls, CRPS).

We did not estimate the centroid of each finger representation because our mapping method is not designed to reveal independent representations of individual fingers, given that each finger is stimulated in succession. Future studies are required to investigate finger-specific representations in CRPS.

##### Evaluation of hand map geometry.

As a measure of the functional geometry of the map, we measured the spatial arrangement (i.e., direction) of the spatial gradients of the map. As illustrated in Figure 7*A*, the hand map exhibits a typical spatial gradient from index finger to little finger. For each participant, we resampled the map ROIs from the inflated cortical surface of each participant onto a flattened, 2D surface patch. After sampling the complex valued 3D phase-mapping data to the folded surface, we displayed it on a small flattened, 2D surface patch, which minimizes deviations from original geometry. We gently smoothed the complex values on the surface using a 1.5 mm kernel and then converted the complex valued data (real, imaginary) to amplitude and phase angle. The 2D gradient of the phase angle was computed after fitting a plane to the data from the surrounding vertices (taking care to circularly subtract the angular data). The amplitude of the gradient at each vertex was then normalized for display.

The mean direction of map gradients is not informative because each participant cortical patch has an arbitrary direction. However, the variability of map gradients is informative because it does not depend on the orientation of the cortical surface patch; higher variability of gradient directions indicates that the map phases are more spread and less spatially organized. Therefore, we investigated whether the functional geometry of the map is affected by CRPS, by testing whether the gradient directions of the map of the affected hand were more variable than those of the unaffected hand and controls (after pooling data from the two CRPS groups). As a measure of map gradient variability, we calculated the circular variance of the gradient angles of each ROI. We conducted a Harrison–Kanji test (Harrison and Kanji, 1988; Berens, 2009) on the gradient variances to statistically compare the variability of map gradients across groups and participants. This test allowed us to perform a two-factor ANOVA for circular data, with a within-subject factor side (two levels: affected, unaffected) and a between-subjects factor group (two levels: controls, CRPS). BFs for each contrast were calculated as described by Equation 4 (the probability level for *H*_0_ was 0.05).

We tested the hypothesis that there was a relation between map gradient variability and disease duration, using the equation for circular-linear correlation (*r*_cl_) described by Zar (1999, their Eq. 27.47). A *p* value for *r*_cl_ is computed by considering the test statistic *N r*_cl,_, which follows a χ^2^ distribution with 2 degrees of freedom (Berens, 2009). BFs based on the χ^2^ distribution were calculated following Equation 4 (with 0.05 probability level for *H*_0_).

##### Data normalization.

We used Shapiro–Wilks tests to evaluate whether the variables were normally distributed. The variables that deviated from the normal distribution were log-transformed; these were measures of map area and geodesic distance from the central sulcus in both hemispheres, pain rating during the scan, *Quick*Dash score, and disease duration. Upon log-transforming these variables, we confirmed that they were normally distributed (again using Shapiro–Wilks tests).

##### Relation with clinical measures.

In the CRPS group, we evaluated whether the map measures we derived from single-subject ROIs (area, centroid location, gradient variability) correlated with six clinical measures: disease duration, the *Quick*Dash score reflecting the severity of upper limb disability, average pain intensity rated in three time windows (during the MRI scans, and in the 2 and 7 d before the scans), and a severity score derived from the difference of PPT thresholds in the two hands as follows:

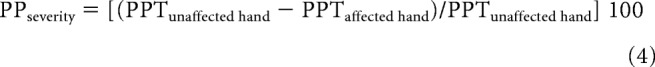
 Disease duration was log-transformed because it was not normally distributed. Pairwise correlation coefficients between clinical measures and map measures for the affected and unaffected hemispheres were Fisher-transformed and compared using a *z* test. The resulting *p* value was compared against a critical *p* value corrected for a 5% false discovery rate using the Benjamini–Hochberg procedure (Hochberg and Benjamini, 1990).

##### Cross-subject average (for illustration).

We averaged maps across subjects purely for illustration. All statistical analyses were performed on measures derived from the individual-subjects maps. We first inflated each participant's cortical surface to a sphere, and then nonlinearly morphed it into alignment with an average spherical cortical surface using FreeSurfer's tool mri_surf2surf (Fischl et al., 1999). This procedure maximizes alignment between sulci (including the central sulcus) while minimizing metric distortions across the surface. Four steps of nearest-neighbor smoothing (<1.5 mm FWHM in 2D) were applied to the data after resampling on the spherical surface. Complex valued mapping signals were then combined across all subjects (independently of whether the S1 map was detected or not) on a vertex-by-vertex basis by vector averaging (Mancini et al., 2012). The amplitude was normalized to 1, which prevented overrepresenting subjects with strong amplitudes. Finally, a scalar cross-subject *F* ratio was calculated from the complex data and rendered back onto fsaverage (uncorrected, *p* < 0.05).

#### Software and data availability

Software to perform phase-mapping analyses is openly available at http://www.cogsci.ucsd.edu/∼sereno/.tmp/dist/csurf. We used an open-source software (JASP) for the Bayesian statistical analyses: https://jasp-stats.org. Each individual hand map ROI is available at https://osf.io/w3zbe/.

## Results

### Demographics and sensitivity to pain

Table 1 reports the demographic and clinical information of the study sample (healthy controls: *n* = 17; CRPS to the left hand: *n* = 8; CRPS to the right hand: *n* = 10). Age was similar in the control group (mean ± SD, 44.9 ± 12.0 years) and in the patient group (44.2 ± 11.3; independent-samples *t* test: *t*_(33)_ = 0.19, *p* = 0.856, BF_10_ = 0.329). Handedness was evaluated using the Edinburgh Handedness Inventory, which yields a laterality score that ranges from −100 (left-hand dominant) to 100 (right-hand dominant) (Oldfield, 1971). This laterality score was comparable in controls (73.6 ± 49.8) and patients (61.6 ± 58.1; independent-samples *t* test: *t*_(33)_ = 0.65, *p* = 0.518, BF_10_ = 0.384). Age of patients was similar to those found in the UK CRPS Registry (Shenker et al., 2015): mean age at onset was 43 ± 12.7 years (*n* = 239), whereas mean pain duration was 2.9 years (*n* = 237), slightly shorter than in the UK CRPS registry.

We found weak evidence that CRPS patients were more sensitive to pressure, with lower average PPT on their affected hand (3.4 ± 3.8) than on their unaffected hand (7.6 ± 11.0; paired-samples *t* test: *t*_(17)_ = −2.21, *p* = 0.041, BF_10_ = 1.679). Confirming that the CRPS was unilateral, PPTs on the unaffected hand of CRPS patients were similar to those of controls (average left and right hand of controls ± SD, 10.7 ± 14.9; independent-samples *t* test: *t*_(33)_ = 0.72, *p* = 0.476, BF_10_ = 0.398). Ratings of spontaneous pain did not vary in a consistent fashion before and after the imaging session (mean difference ± SD, 0.6 ± 2.5; *t*_(16)_ = 0.96, *p* = 0.351, BF_10_ = 0.281).

### Somatotopic representation of the hand in S1

**Figure 2.**
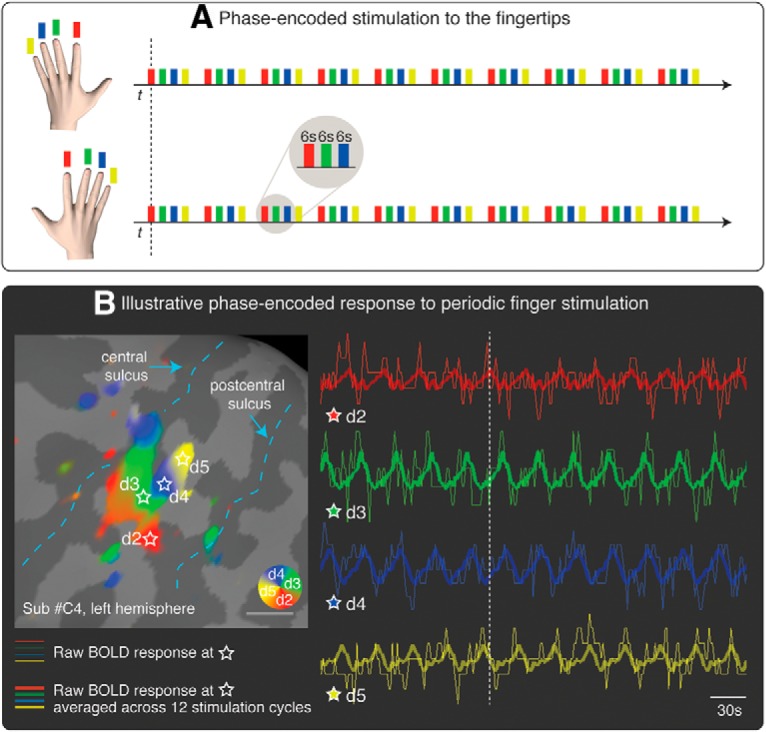
***A***, Phase-encoded stimulation procedure. The tip of the index finger (red, d2), middle finger (green, d3), ring finger (blue, d4), and little finger (yellow, d5) was stimulated in succession, in repeated cycles (12 cycles per run). To reduce scanning time, the homologous fingers of the right and left hands were stimulated simultaneously. ***B***, Illustrative phase-encoded response to periodic fingertip stimulation. The figure shows the raw BOLD response in four voxels of interest (thin lines; data were motion-corrected and the linear trend removed). The locations of the voxels are marked with a star on the cortical surface of the left primary somatosensory cortex of 1 participant. Thicker lines indicate the average of the raw BOLD response across 12 cycles of stimulation. The vertical, dashed, white line is displayed to facilitate the visualization of the shift of the phase of the BOLD response across the four voxels. The *F* statistics of the signal at different phases are rendered on the inflated cortical surface and color-coded as in ***A*** (cluster-corrected *p* < 0.01). Phases corresponding to rest have been truncated.

**Figure 3.**
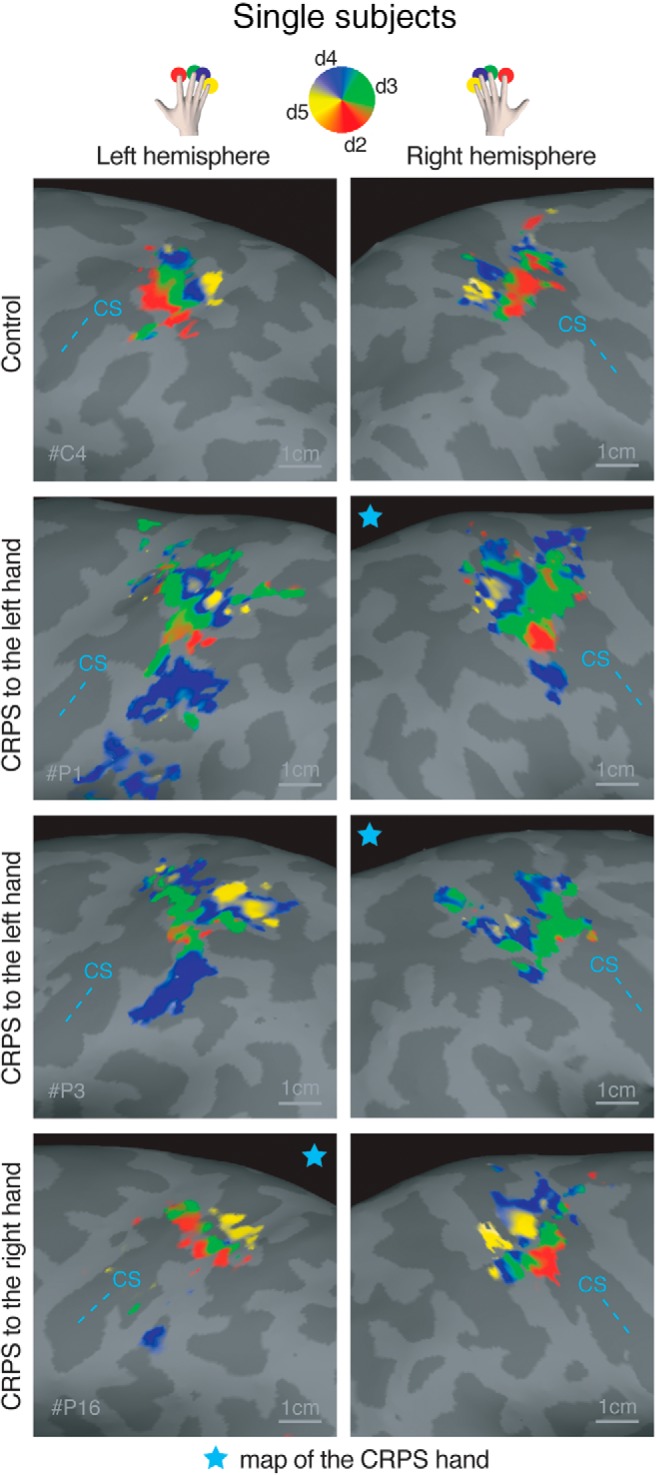
Phase maps of the hand in an illustrative control participant and 3 CRPS patients. Top, Color-coding scheme: red represents d2; green represents d3; blue represents d4; yellow represents d5. Phases corresponding to rest have been truncated. Statistical thresholding and cluster correction at *p* < 0.01 were applied to each individual-participant data. CS, Central sulcus. Star represents the map of the CRPS hand.

**Figure 4.**
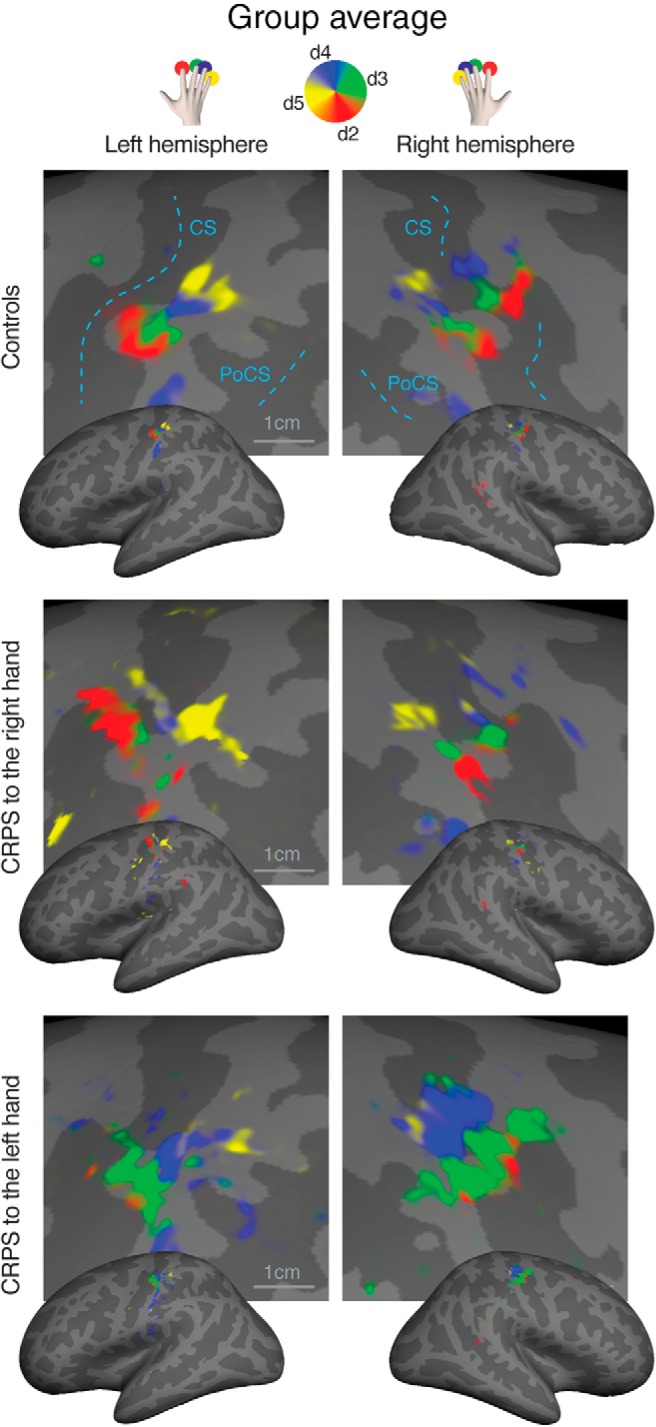
Surface-based average of phase maps in controls, patients with CRPS to the right hand, and patients with CRPS to the left hand. The complex valued mapping data were averaged in a spherical surface coordinate system after morphing each subject's data into alignment with an average spherical sulcal pattern, and the *F* statistics were rendered back onto an average unfolded cortical surface (Freesurfer's fsaverage, inflated_average; uncorrected *p* < 0.05 only for illustration). Top, The color-coding scheme is as follows: red represents d2; green represents d3; blue represents d4; yellow represents d5. Phases corresponding to rest have been truncated. CS, Central sulcus; PoCS, postcentral sulcus.

We stimulated the tips of each finger in succession, as shown in Figure 2*A*, using a mechanical probe. Mechanical stimulation to the fingertips elicited a periodic response in the hand region of S1 (Fig. 2*B*). A selection of single-subjects maps is shown in Figure 3, and the average maps are displayed in Figure 4. The map phase angle (indicating finger preference) is displayed using a continuous color scale (red to green to blue to yellow), the saturation of which is masked by the statistical threshold. All analyses were performed on individual subject data (cluster-corrected at *p* < 0.01), but uncorrected group maps (*p* < 0.05) are displayed in Figure 4 merely for illustration. Phases corresponding to rest (no stimulation) have been truncated. The map showed a clear spatial gradient of digit preference, progressing from d2 (index finger) to d3, d4, and d5 (little finger). The arrangement and location of the map were qualitatively similar to that reported in previous human fMRI studies (Sanchez-Panchuelo et al., 2010; Mancini et al., 2012; Besle et al., 2013; Martuzzi et al., 2014; Kolasinski et al., 2016a).

We tested whether the area, location, and functional geometry of the map of the affected hand were similar to those of the unaffected hand and controls. To do so, we defined individual ROIs as clusters located in S1 that showed a significant periodic response at the spatial frequency of stimulation (cluster-corrected, *p* < 0.01).

### Map area

**Figure 5.**
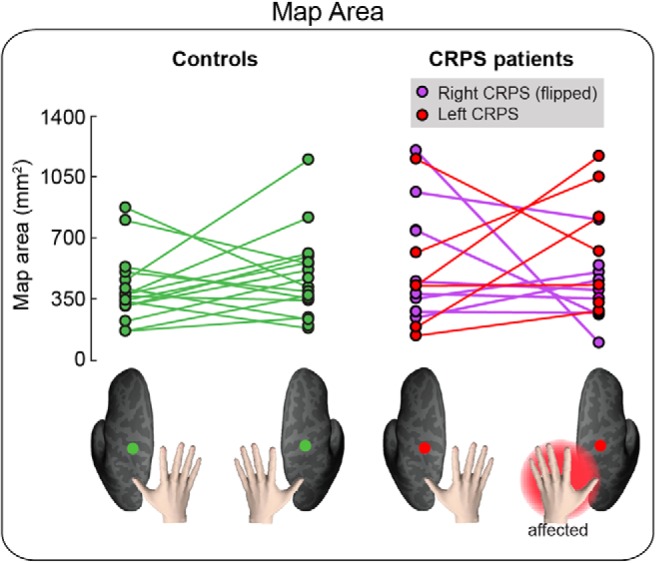
Area of the hand map in S1. The area of the hand map (in mm^2^) in the left hemisphere and right hemisphere is plotted for each group and individual participant. To facilitate comparison, data from the two CRPS groups (right hand CRPS, left hand CRPS) were pooled, after flipping the data from one group (right hand CRPS) so that the affected side is always the left hand/right hemisphere in all patients.

To control for interindividual variability in brain size, we resampled the phase maps onto the original average brain volume. We then calculated the surface area of the left- and right-hand maps from each participant ROI. As evident in Figure 5*A*, the map area was comparable among groups and sides. A mixed-effects ANOVA with a within-subject factor side (two levels: affected, unaffected) and a between-subjects factor group (two levels: controls, CRPS) did not provide evidence for any main effect or interaction (side: *F*_(1,28)_ = 0.404, *p* = 0.530, η^2^ = 0.007; group: *F*_(1,28)_ = 0.499, *p* = 0.486, η^2^ = 0.017; side × group: *F*_(1,28)_ = 0.303, *p* = 0.586, η^2^ = 0.005). A Bayesian mixed-effects ANOVA was inconclusive; it provided stronger (although overall weak) evidence for the null model (BF_10_ = 1, P(M|data) = 0.532) relative to models of group (BF_10_ = 0.376, P(M|data) = 0.200), side (BF_10_ = 0.331, P(M|data) = 0.176), side+group (BF_10_ = 0.124, P(M|data) = 0.066), and side+group+interaction (BF_10_ = 0.051, P(M|data) = 0.027). Given that the Bayesian ANOVA was inconclusive, we conducted a follow-up Bayesian independent-sample *t* test on the map area averaged across hemispheres; there was no evidence for a difference in map area between CRPS patients and healthy volunteers (BF_10_ = 0.424, error 0.013%). As a further check, we confirmed with Bayesian paired-samples *t* tests that, in the CRPS group, the map area was comparable across hemispheres (BF_10_ = 0.270, error 0.008%).

In summary, these analyses do not provide support for the hypothesis that the map of the CRPS hand was smaller than the map of the unaffected hand and that of healthy controls, at group level.

### Map location

**Figure 6.**
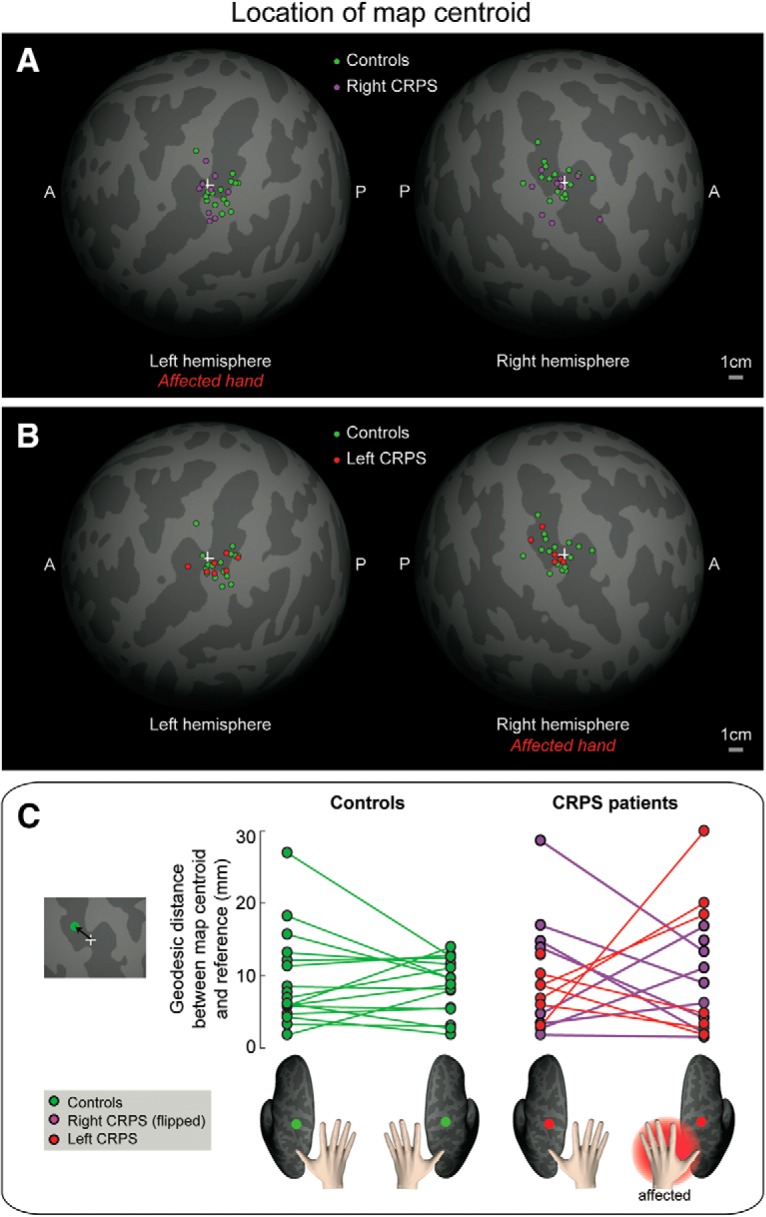
***A***, ***B***, Spatial distribution of map centroids. The location of the centroid of the hand map in each individual subject is displayed on an average spherical cortical surface. White cross represents an arbitrary reference point on the central sulcus. ***C***, Geodesic distance (in millimeters) between each map centroid and a reference point (+) on the central sulcus. To facilitate comparison, data from the two CRPS groups (right hand CRPS, left hand CRPS) were pooled, after flipping the data from one group (right hand CRPS) so that the affected side is the left hand/right hemisphere in all patients.

We calculated the centroid of the hand map, after resampling it onto an average spherical surface (for details, see Evaluation of hand map location in *Statistical analysis* section.). This was done to control for individual differences in brain morphology and to obtain localization measures that were not confounded by gyrification. Figure 6*A*, *B* shows the distribution of map centroids of each participant, resampled onto a canonical spherical cortical surface of an average brain; the map centroid location was variable among participants of each group, but visibly similar across groups. Indeed, the *F* statistics based on the Fisher probability density function (Fisher, 1953) did not provide evidence for any directional difference between groups for either side (Table 2).

**Table 2.** Statistical values for the comparison of the locations of map centroids between groups

Contrast	Side	*F*	df	*p*	BF_10_
Controls versus right CRPS	Left hemisphere	0.002	2,50	0.998	0.001
Controls versus right CRPS	Right hemisphere	0.025	2,48	0.975	0.306
Controls versus left CRPS	Left hemisphere	0.005	2,42	0.995	0.309
Controls versus left CRPS	Right hemisphere	0.001	2,44	0.999	0.311

As a further comparison of the locations of map centroids across groups, we computed the geodesic distance (in millimeters) between the map centroid and an arbitrary reference point located within the concavity of the central sulcus (Fig. 6*C*). Importantly, geodesic distance measures calculated onto average spherical surfaces are not confounded by gyrification and allow comparison of different subjects. This is a key advantage of our approach over previous studies, which measured Euclidean distances between two finger representations. A mixed-effects ANOVA with a within-subject factor side and a between-subjects factor group did not provide evidence for any main effect or interaction (side: *F*_(1,28)_ < 0.01, *p* = 0.890, η^2^ < 0.001; group: *F*_(1,28)_ = 0.125, *p* = 0.727, η^2^ = 0.004; side × group: *F*_(1,28)_ < 0.01, *p* = 0.974, η^2^ < 0.001). In a Bayesian mixed-effects ANOVA, the null model had stronger evidence (BF_10_ = 1, P(M|data) = 0.576) than the models of group (BF_10_ = 0.352, P(M|data) = 0.203), side (BF_10_ = 0.263, P(M|data) = 0.151), side+group (BF_10_ = 0.091, P(M|data) = 0.052), and side+group+interaction (BF_10_ = 0.031, P(M|data) = 0.018). The follow-up Bayesian independent-sample *t* test on the map centroid location averaged across hemispheres did not provide evidence for a difference between groups (BF_10_ = 0.325, error 0.011%). In the CRPS group, the centroid location was comparable across hemispheres (BF_10_ = 0.278, error 0.009%).

Together, these analyses indicate that the location of the hand map centroid was not affected by CRPS.

### Map geometry

**Figure 7.**
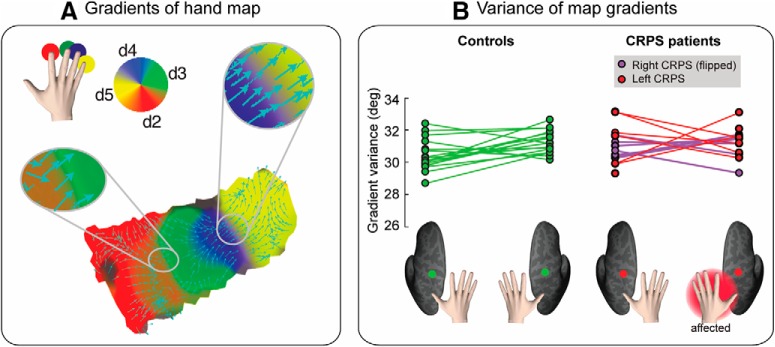
***A***, Gradients of the hand map. Gradients of a single-subject phase map are displayed as cyan arrows over a flattened (2D) cortical surface patch. The gradient points in the direction of the greatest rate of increase of the function (i.e., the direction of the phase shift in the hand map). Color-coding scheme of the hand map is as follows: red represents d2; green represents d3; blue represents d4; yellow represents d5. ***B***, Variability of hand map gradients. The circular variance of map gradient directions is displayed for each participant and condition (side: left hemisphere, right hemisphere; group: controls, CRPS patients). Bottom, The color-coding scheme. To facilitate comparison, data from the two CRPS groups (right hand CRPS, left hand CRPS) were pooled, after flipping the data from one group (right hand CRPS) so that the affected side is the left hand/right hemisphere in all patients.

Finally, we evaluated the variability of the geometry of the map of the affected hand in CRPS patients. As illustrated in Figure 7*A*, the hand map exhibits a typical spatial gradient from index finger to little finger. The spatial gradient (i.e., the direction) of the map indicates the spatial progression of the map phases, providing a measure of the map geometry. We investigated whether the gradient directions of the map of the affected hand were more variable than those of the unaffected hand and controls. As a measure of map gradient variability, we calculated the circular variance of the gradient angles of each flattened, 2D, surface ROI (for details, see Evaluation of hand map geometry in *Statistical analysis* section).

The gradient directions of the map of the affected hand were not differently variable (i.e., not differently spread) from those of the unaffected hand and controls (Fig. 7*B*). A Harrison–Kanji test with a within-subject factor side and a between-subjects factor group on the gradient variances provided weak evidence for a main effect of side (*F*_(1,59)_ = 4.813, *p* = 0.032, _p_η^2^ = 0.079, BF_10_ = 1.202) and no evidence for a main effect of group (*F*_(1,59)_ = 2.243, *p* = 0.140, _p_η^2^ = 0.038, BF_10_ = 0.560). We found weak and inconclusive evidence for an interaction between side and group (*F*_(1,59)_ = 3.889, *p* = 0.071, _p_η^2^ = 0.057, BF_10_ = 0.971). This suggests that the spread of map gradients, which is a measure of functional organization, was largely similar across groups.

### No relation with clinical measures

We found no evidence for a differential relation between affected versus unaffected hand map measures (area, location, and geometry) and clinical measures reflecting disease duration and severity (pain intensity measured in different time windows, PP_severity_, upper limb disability). The results of these analyses are reported in Table 3. We do not interpret correlations for one side (e.g., affected hand map) independently of the other (e.g., unaffected hand map) (Nieuwenhuis et al., 2011).

**Table 3.** Relation between hand map (area, centroid location, gradient variability) and clinical measures (CRPS duration, quickDASH score of upper limb disability, average pain intensity rated during the MRI scans, in the 2 and 7 d preceding the MRI session, and severity score derived from pain pressure thresholds)*^a^*

Variable 1	Variable 2	*r* (affected side)	*r* (unaffected side)	*z* test	*p* (two-tailed)	Critical B-H (5% FDR)
Map area	CRPS duration	−0.56	−0.13	−1.26	0.208	0.017
Map area	Limb disability score	0.06	0.00	0.15	0.881	0.033
Map area	Pain rating (during scan)	0.20	0.23	−0.08	0.936	0.042
Map area	Pain rating (2 d)	0.55	−0.02	1.63	0.103	0.008
Map area	Pain rating (7 d)	−0.21	0.02	−0.60	0.549	0.025
Map area	PP severity	−0.31	−0.34	0.07	0.944	0.050
Map location	CRPS duration	−0.19	0.52	−1.98	0.048	0.008
Map location	Limb disability score	−0.03	0.02	−0.15	0.881	0.033
Map location	Pain rating (during scan)	0.30	−0.01	−0.74	0.459	0.017
Map location	Pain rating (2 d)	0.31	0.14	0.47	0.638	0.025
Map location	Pain rating (7 d)	−0.06	−0.04	−0.03	0.976	0.042
Map location	PP severity	0.01	0.01	−0.02	0.984	0.050
Map gradient variance	CRPS duration	0.40	0.20	0.55	0.5823	0.025
Map gradient variance	Limb disability score	0.35	0.22	0.37	0.7114	0.033
Map gradient variance	Pain rating (during scan)	0.28	0.26	0.07	0.9442	0.050
Map gradient variance	Pain rating (2 d)	0.17	0.24	−0.19	0.8493	0.042
Map gradient variance	Pain rating (7 d)	0.31	0.63	−1.03	0.303	0.008
Map gradient variance	PP severity	0.52	0.21	0.90	0.3681	0.017

*^a^*Pairwise correlations were performed between the variables listed in the first and second columns, separately for the affected and unaffected hemispheres (patients only). The resulting Pearson's r coefficients are reported in columns 3 and 4, transformed in *z* scores, and compared using a *z* test; *z* scores and uncorrected *p* values for these comparisons are reported in columns 5 and 6. The FDR-corrected critical *p* value (using a Benjamini–Hochberg procedure) is reported in column 7.

## Discussion

We show that the cortical map of the fingertips of the CRPS hand in S1 is strikingly comparable with the map of the unaffected hand and controls in terms of area, location, orientation, and geometry. Our results do not exclude that other abnormalities may occur at S1 level, such as excitability changes (Lenz et al., 2011; Di Pietro et al., 2013), morphological (Baliki et al., 2011; Pleger et al., 2014; compare van Velzen et al., 2016), and connectivity changes (Geha et al., 2008). However, our findings challenge or, at the very least, narrow the notion of S1 map reorganization in CRPS. Thus, even if a map reorganization occurs, it does not appear to be directly related to pain severity and upper limb disability.

These findings urge us to reconsider the mechanisms that are currently proposed to underpin CRPS (Marinus et al., 2011). They also compel us to reevaluate the rationale for clinical interventions that aim to reduce pain by “restoring” somatotopic representations with sensory discrimination training (Moseley et al., 2008b; Catley et al., 2014), or by correcting sensorimotor incongruences (which are thought to be induced by S1 reorganization) with mirror therapy (McCabe et al., 2003; but see Moseley and Gandevia, 2005; Moseley et al., 2008b). Although these interventions appear to offer clinical benefit (O'Connell et al., 2013), they are unlikely to engender a “restoration” of somatotopic representations in S1, which are largely comparable to those of controls.

### Revisiting previous evidence of somatotopic reorganization in CRPS

Comparisons across different studies are inevitably challenging due to the complexity and variety of CRPS symptomatology; in previous studies, patients varied greatly in regard to the combination, severity, and duration of their symptoms. Our study suggests that map size is probably related to disease duration, although only a longitudinal study could confirm a causal relationship.

Our results also show how important it is to consider methodological issues when using functional neuroimaging to understand the pathophysiology of clinial pain conditions. The notion of somatotopic reorganization in CRPS was mostly based on studies that used imaging methods (EEG/MEG) with lower spatial resolution than fMRI (Juottonen et al., 2002; Maihöfner et al., 2003; Pleger et al., 2004; Vartiainen et al., 2008, 2009). A more recent study used fMRI and measured the cortical distance between d1 and d5 activation peaks (Di Pietro et al., 2015). This study partially confirmed former EEG/MEG findings, reporting that the d1-d5 distance in S1 was smaller for the affected hand than it was for the unaffected hand in CRPS patients. However, the representation of the affected hand was comparable to that of healthy controls, in agreement with the current results. Critically, Di Pietro et al. (2015) found that the representation of the unaffected hand in CRPS patients was larger than that of controls, thus challenging the view that the representation of the affected hand is shrunk and suggesting that the representation of the unaffected hand is actually enlarged. The current results do not support either interpretation.

Three important limitations affect all previous studies, regardless of the imaging approach used. First, the approach taken to estimate map size is both indirect and incomplete because it is based on the measurement of the Euclidean distance between the activation maxima of two fingers (d1 and d5). Instead, the area of the map of all fingers is a more direct and complete measure of map size. Second, Euclidean measures of cortical distances can be inaccurate because they disregard that the cortical surface is not flat, especially in the regions of the sulci. Third, Euclidean distance measures can be affected by nontopographical, structural changes in S1, which can be associated with CRPS (Baliki et al., 2011; Pleger et al., 2014). The latter two problems can be overcome by morphing activation maps onto a reconstruction of the flattened cortical surface (Makin et al., 2013a; Kikkert et al., 2016), but previous studies on CRPS patients have not taken this approach. Together, these methodological issues can affect both the accuracy and validity of previous measures of map extent.

### Stability of cortical topographies

Recent fMRI studies (Makin et al., 2013a; Kikkert et al., 2016) suggest that finger topographies in S1 are surprisingly persistent, even in humans who suffered amputation of the upper limb. It was demonstrated that the area, location, and functional organization of the S1 maps of the missing hand were similar, although noisier, to those observed in controls during finger movements (Makin et al., 2013a; Kikkert et al., 2016). It has also been shown that the deafferented territory in human S1 can respond to somatotopically adjacent body regions (i.e., the lip for upper limb amputees) (Flor et al., 1995; Flor, 2008), or to body regions that the amputees overuse to supplement lost hand function (e.g., the intact hand). This results in a highly idiosyncratic remapping, which does not necessarily involve adjacent representations in S1 (Makin et al., 2013b; Philip and Frey, 2014). Thus, cortical reorganization in amputees is not dictated by cortical topographies but can depend on compensatory use of other body parts. Similarly, short-term shifts in S1 maps can occur in healthy participants after surgical gluing of the index and middle fingers for 24 h. These changes are thought to depend on compensatory use of the fourth and fifth fingers (Kolasinski et al., 2016b). These studies support the view that the S1 changes previously reported in CRPS patients might not directly related to pain, but it remains to be determined why map shrinkage relates to disease duration. Could map size be related to hand use? We found no relation between map size and severity of the upper limb disability.

Recent evidence from electrophysiological and inactivation studies in monkeys suggests that the reorganization following nerve transection originates, not in S1, but in the brainstem. Indeed, inactivating the cuneate nucleus abolishes the neural activity in the deafferented limb representation in S1 elicited by face stimulation (Kambi et al., 2014). Hence, loss of input from a body region in adulthood may lead to the formation or potentiation of lateral connections in the brainstem, which gives rise to a new pathway from periphery to cortex. It is not clear whether this new pathway contributes to cortical reorganization, but the original pathway seems to be relatively spared, even under the extreme circumstance of limb amputation (Makin and Bensmaia, 2017).

Some resistance to change has also been described for visual retinotopic maps. Although it has been shown that large lesions to the retina in adult mammals can induce a reorganization of retinotopic cortical maps in primary visual cortex (Kaas et al., 1990), more recent studies have reported that the topography of the macaque primary visual cortex does not change (for at least 7 months) following binocular retinal lesions (Smirnakis et al., 2005). Similarly, severe eye diseases, such as retinal degeneration, do not seem to affect retinotopic representations in the human early visual cortex (Xie et al., 2012; Haak et al., 2016). Together, these findings suggest that cortical topography is more stable and resistant to change than what it was initially thought.

### Conclusion and future directions

Our study provides the most complete characterization, to date, of the S1 somatotopy of the CRPS hand. We report that the S1 representation of the CRPS hand is comparable, at the group level, to that of the healthy hand, in terms of cortical area, location, and geometry. The phase-mapping methods we used are not suitable to evaluate finger-specific representations and their level of overlap. Future studies using randomized stimulation are required to evaluate whether the degree of overlap between individual finger representations (Ejaz et al., 2015) is affected in CRPS patients. Moreover, future longitudinal studies are required to determine how the map changes over time and its effect on sensorimotor function.
